# Diversity of 2D Acoustofluidic Fields in an Ultrasonic Cavity Generated by Multiple Vibration Sources

**DOI:** 10.3390/mi10120803

**Published:** 2019-11-22

**Authors:** Qiang Tang, Song Zhou, Liang Huang, Zhong Chen

**Affiliations:** 1 Faculty of Mechanical and Material Engineering, Huaiyin Institute of Technology, Huaian 223001, China; zs41080218@126.com (S.Z.); chenzhong@hyit.edu.cn (Z.C.); 2 School of Instrument Science and Opto-Electronics Engineering, Hefei University of Technology, Hefei 230009, China; lianghuang@hfut.edu.cn

**Keywords:** acoustic streaming, diversity, acoustofluidic field, Matryoshka structure

## Abstract

Two-dimensional acoustofluidic fields in an ultrasonic chamber actuated by segmented ring-shaped vibration sources with different excitation phases are simulated by COMSOL Multiphysics. Diverse acoustic streaming patterns, including aggregation and rotational modes, can be feasibly generated by the excitation of several sessile ultrasonic sources which only vibrate along radial direction. Numerical simulation of particle trajectory driven by acoustic radiation force and streaming-induced drag force also demonstrates that micro-scale particles suspended in the acoustofluidic chamber can be trapped in the velocity potential well of fluid flow or can rotate around the cavity center with the circumferential acoustic streaming field. Preliminary investigation of simple Russian doll- or Matryoshka-type configurations (double-layer vibration sources) provide a novel method of multifarious structure design in future researches on the combination of phononic crystals and acoustic streaming fields. The implementation of multiple segmented ring-shaped vibration sources offers flexibility for the control of acoustic streaming fields in microfluidic devices for various applications. We believe that this kind of acoustofluidic design is expected to be a promising tool for the investigation of rapid microfluidic mixing on a chip and contactless rotational manipulation of biosamples, such as cells or nematodes.

## 1. Introduction

Contactless manipulation of micro-/nano-scale particles and biosamples in a microfluidic chamber is of great importance [1,2,3] due to the increasing requirement to carry out elementary operations like trapping [4,5], rotation [6,7], separation [8], and concentration [9], which are an essential technique for biological researches and exhibit numerous commercial applications in the bioengineering and pharmaceutical industries [10,11,12]. Various microfluidic manipulation methods for capturing [13,14], transporting [15], rotating [16], and separating [17] of micro-/nano-scale objects have been provided and verified in laboratories worldwide. The core issue of microfluidic manipulation is to generate enough driving forces that can overcome the viscous resistance forces caused by the surrounding media or the adhesive effects at the solid-fluid interfaces, especially for biosamples [18]. Conventional active manipulation methods of micro-/nano-scale particles and biosamples include electric/dielectric [19], magnetic/diamagnetic [20], optical [21], and acoustic effects [22], taking advantage of powerful driving forces and relatively high manipulation accuracy. Existing passive manipulation techniques involve particle filtration [23], inertial movement [24], and hydraulic driving [25], taking advantage of simple device fabrication and relatively high throughput. 

Among the aforementioned manipulation methods, acoustic methods, including acoustic tweezers and cavities based on acoustic radiation force and Stokesian drag forces generated by acoustic streaming fields, are often considered to have the merits, such as little physiological damage to manipulated bio-samples (biocompatibility) [26], contactless manipulation in a non-invasive way [27], low dependence on electromagnetic and optical properties of samples [28], simple and low-cost microfluidic platforms [29], and so on. Acoustic radiation force arises from the interaction of ultrasonic fields with particles when there are acoustic property differences between particles and media, while acoustic streaming is commonly caused by ultrasonic energy absorption in viscous media. Unlike the acoustic radiation force, acoustic streaming is a steady fluid motion and can be generated without the existence of particles in the acoustofluidic field [30,31]. Generally speaking, there are two main kinds of acoustic streaming [1,2]: One is the boundary layer driven acoustic streaming, which is formed by the acoustic energy dissipation into the viscous boundary layer of a fluid along any solid boundary that is comparable or greater in length (in the acoustic propagation direction) than a quarter of the acoustic wavelength [32,33], the other is the bulk wave driven acoustic streaming, which is the flow formed by the dissipation of acoustic energy into the bulk of a fluid and is essential for three-dimensional acoustic streaming analysis and micro-/nano-particle manipulation [34,35,36]. In recent years, diverse research of acoustic streaming patterns has gained increasing attention [37,38]. Since the Reynolds stress force in the Navier–Stokes equation arises from the nonlinear time-average terms over a one time period, the acoustic streaming pattern is dramatically affected by the distribution, frequency, amplitude, and phase differences of multiple incident sound sources, the acoustic and fluidic properties of different media, and the device structure design, including the shape and distribution of fluid–solid interfaces [1,39,40].

According to existing literatures and our published papers [37,41,42,43], counter-rotating acoustic streaming vortices generated by only one vibration source usually come in pairs if the initial flow field is static. This is because the fluidic viscosity outside the viscous boundary layer is relatively small or negligible compared with that inside the boundary layer, so the conservation law of vorticity needs to be satisfied. In this paper, by referring to the structural design schemes proposed by Bernassau et al. (2014) [28] and Courtney et al. (2019) [44], a novel method to generate an abundant of acoustic streaming vortices only using radial vibration of segmented ring-shaped vibration sources in an acoustofluidic cavity is proposed, in which the height of the fluidic medium is negligible in comparison with other dimensions. Thus, a two-dimensional (2D) acoustofluidic model can be used in our simulation despite the fact that it belongs to a bulk wave device in engineering. The differences in structure design only lie in the number, space distribution, and initial phase change of vibration sources. With the change of phase differences among multiple vibration sources, diverse acoustic streaming patterns can be realized with ease. According to the simulation results of the particle tracking module, the distribution of the petal-like acoustofluidic field can not only accumulate micro-particles to a low acoustic streaming speed area but can also drive particles to rotate along a clockwise or counterclockwise direction under the influence of circumferential flow. Inspired by Elford et al. 2011 [45], we also designed simple Russian doll- or Matryoshka-type configurations with double-layer vibration sources. Fragmentation and separation of acoustic streaming vortices can be feasibly available only by changing initial phases of multiple vibration sources. Ample structural designs on the combination of phononic crystals and acoustic streaming fields can be provided in the following research. However, unlike the ultrasonic field, which can be distributed in the whole microfluidic chamber, the acoustic streaming field is commonly restrained in the acoustofluidic cavity composed of the multiple vibration sources. This platform design of segmented ring-shaped vibration sources offers flexibility for the control of acoustic streaming fields in microfluidic devices for various applications, which is expected to be a promising tool for the investigation of generation mechanisms of rotational acoustic streaming [46], rapid microfluidic mixing of different media or particles on a chip [47], and non-contact manipulation and rotation of bio-samples [48] for the following surface morphology observation and phenotype characterization [49].

## 2. Theory and Simulation

The fundamental governing equations of acoustic streaming generated by acoustically oscillating sources in a finite chamber have been studied extensively in the literature [50,51,52]. Here, we briefly introduce the perturbation theory and a relevant simulation process for completeness. Sound energy emitted from the vibration sources into a viscous medium and boundary layer is nonlinear. This effect can cause a time-independent, steady fluid flow, known as acoustic streaming in a microfluidic chamber [32]. Here, a homogeneous isotropic fluid is assumed, in which the continuity and momentum equations for the fluid flow can be expressed as:

(1) $$\frac{\partial \rho }{\partial t}+\nabla \cdot (\rho u)=0$$

(2) $$\rho (\frac{\partial u}{\partial t}+u\cdot \nabla u)=-\nabla p+\mu \nabla^{2}u+(\mu_{b}+\frac{1}{3}\mu )\nabla \nabla \cdot u$$

where *ρ* is the fluid density, *t* is time, **u** is the fluid velocity vector, *p* is the pressure, and *μ* and *μ_b_* are the dynamic and bulk viscosity coefficients of the fluid, respectively. The left-hand side of Equation (2) represents the inertial force per unit volume on the fluid, with the two terms in the bracket being the unsteady acceleration and convective acceleration of a fluid volume element, respectively. The right-hand side indicates the divergence of stress, including the pressure gradient and viscosity forces. Other forces, such as the gravity force, are not considered because they are generally negligible compared to the above-mentioned driving forces [53]. In the case of small oscillation amplitude, the induced fluidic response can be expressed using a perturbation expansion, and the fluid density, pressure, and velocity are written as:

(3) $$\rho =\rho_{0}+\rho_{1}+\rho_{2}+\cdots$$

(4) $$p=p_{0}+p_{1}+p_{2}+\cdots$$

(5) $$u=u_{1}+u_{2}+\cdots$$

where the subscripts 0, 1, and 2 represent the static (absence of sound), first-order, and second-order quantities, respectively. The unwritten higher order terms in Equations (3)–(5) can be ignored in the following calculation of first-order sound field and second-order acoustic streaming field because of the higher order infinitesimal quantities [50]. Substituting Equations (3)–(5) into Equations (1) and (2) and considering the first-order terms can yield:

(6) $$\frac{\partial \rho_{1}}{\partial t}+\rho_{0}\nabla \cdot u_{1}=0$$

(7) $$\rho_{0}\frac{\partial u_{1}}{\partial t}=-\nabla p_{1}+\mu \nabla^{2}u_{1}+(\mu_{b}+\frac{1}{3}\mu )\nabla \nabla \cdot u_{1}$$

Repeating the above procedure for the second-order terms, followed by time averaging over a period of oscillation, yields a second-order acoustic streaming field:

(8) $$\bar{\frac{\partial \rho_{2}}{\partial t}}+\rho_{0}\nabla \cdot \bar{u_{2}}+\nabla \cdot \bar{\rho_{1}u_{1}}=0$$

(9) $$\rho_{0}\bar{\frac{\partial u_{2}}{\partial t}}+\bar{\rho_{1}\frac{\partial u_{1}}{\partial t}}+\rho_{0}\bar{u_{1}\cdot \nabla u_{1}}=-\nabla \bar{p_{2}}+\mu \nabla^{2}\bar{u_{2}}+(\mu_{b}+\frac{1}{3}\mu )\nabla \nabla \cdot \bar{u_{2}}$$

where the upper bar represents a time-averaged value over a full oscillation time period. For the steady second-order acoustic streaming field, $\bar{\frac{\partial \rho_{2}}{\partial t}}=0$ and $\bar{\frac{\partial u_{2}}{\partial t}}=0$, Equations (8) and (9) can be simplified as:

(10) $$\rho_{0}\nabla \cdot \bar{u_{2}}=-\nabla \cdot \bar{\rho_{1}u_{1}}$$

(11) $$-\nabla \bar{p_{2}}+\mu \nabla^{2}\bar{u_{2}}+(\mu_{b}+\frac{1}{3}\mu )\nabla \nabla \cdot \bar{u_{2}}=\rho_{0}\bar{u_{1}\cdot \nabla u_{1}}+\bar{\rho_{1}\frac{\partial u_{1}}{\partial t}}$$

where $\bar{u_{2}}=u_{2}$ and $\bar{p_{2}}=p_{2}$ are the time-independent acoustic streaming velocity and pressure needed to be calculated, respectively. Thus, Equations (10) and (11) can also be written as:

(12) $$\rho_{0}\nabla \cdot u_{2}=-\nabla \cdot \bar{\rho_{1}u_{1}}$$

(13) $$-\nabla p_{2}+\mu \nabla^{2}u_{2}+(\mu_{b}+\frac{1}{3}\mu )\nabla \nabla \cdot u_{2}=\rho_{0}\bar{u_{1}\cdot \nabla u_{1}}+\bar{\rho_{1}\frac{\partial u_{1}}{\partial t}}$$

Combining Equations (6), (7), (12), and (13) with appropriate boundary conditions, sound fields and acoustic streaming fields produced by multiple vibration sources can be solved numerically using the commercial finite element software, COMSOL Multiphysics (version 5.4). The computational process consists of the following three steps.

In the first step, the first-order acoustic pressure and velocity field produced by the oscillation of multiple vibration sources was calculated by the ‘thermoviscous acoustics, frequency domain’ module in the COMSOL Multiphysics. Boundary conditions of the sound field were as follows: the vibrating amplitudes and initial phases of multiple sound sources were set by users; the rest of the acoustic boundaries were set to be isothermal. Equations (6) and (7) were used in the calculation of the first-order sound field.

In the second step, computed vibration velocity and sound pressure of the sound field were used to calculate the mass source term $-\nabla \cdot \bar{\rho_{1}u_{1}}$ of Equation (12) and the volume force term $\rho_{0}\bar{u_{1}\cdot \nabla u_{1}}+\bar{\rho_{1}\frac{\partial u_{1}}{\partial t}}$ of Equation (13), respectively, by the post-processing functions of COMSOL Multiphysics, which act as the driving force of acoustic streaming field in an acoustofluidic chamber.

In the last step, the steady acoustic streaming was solved by the fluidic dynamics module ‘laminar flow’ of COMSOL Multiphysics, and the inertial term (Stokes flow) of the fluid flow could be neglected, for the reason that the inertial force $\rho_{0}(u_{2}\cdot \nabla )u_{2}$ was usually negligible, compared with the mass source term and the volume force term in a low-speed acoustic streaming field [52]. Equations (12) and (13) were used in the calculation of acoustic streaming, and all of the fluidic boundaries were set to be no-slip boundary conditions. In order to ensure the convergence of the computational results, weak contributions of mass source and acoustic streaming pressure were added in the fluidic dynamics module [33].

On the basis of the simulated ultrasound field and acoustic streaming field, a COMSOL ‘particle tracing for fluid’ interface can be added to compute the motion of microparticles in a background fluid field. Particle motion in the microfluidic chamber with multiple vibration sources is mainly driven by acoustic radiation force **F**_rad_ (acoustophoretic force) and Stokesian drag force **F**_drag_ [54,55,56], which can be expressed as:

(14) $$F_{\mathrm{rad}}=-\frac{4}{3}\pi R_{p}^{3}\nabla [\frac{1-\beta }{2\rho_{0}c_{0}^{2}}\bar{p_{1}^{2}}-\frac{D}{2}\rho_{0}\bar{{\left\|u_{1}\right\|}^{2}}]$$

(15) $$F_{\mathrm{drag}}=6\pi \mu R_{p}(u_{2}-u_{p})$$

where *R*_p_ and **u**_p_ are the spherical particle radius and velocity, respectively. The parameters *β* and *D* are defined as:

(16) $$\beta =\frac{\rho_{0}c_{0}^{2}}{\rho_{p}c_{p}^{2}}$$

(17) $$D=\frac{3(\rho_{p}-\rho_{0})}{2\rho_{p}+\rho_{0}}$$

where *ρ*_p_ and *c*_p_ are the density and sound speed of the spherical particle, respectively. From the acoustic radiation force and streaming-induced drag force that have been calculated, neglecting the buoyancy force and gravity force of microparticles in a microfluidic chamber, particle trajectories can be modelled, following Newton’s second law of motion [57].

(18) $$\frac{d}{dt}(\rho_{p}\frac{4}{3}\pi R_{p}^{3}u_{p})=F_{\mathrm{rad}}+F_{\mathrm{drag}}$$

In addition to the above-mentioned main driving forces (**F**_rad_ and **F**_drag_), a particle–particle interaction force was also considered in the particle trajectory simulation process to avoid neighboring particles being concentrated to a single point. The particle–particle interaction force can be expressed as:

(19) $$F_{\text{p-p}}=-k_{s}\sum_{j=1}^{N}(\left|r-r_{j}\right|-r_{0})\frac{r-r_{j}}{\left|r-r_{j}\right|}$$

where *k_s_* is the spring constant, **r***_j_* is the position vector of the *j*th particle, and *r*_0_ is the equilibrium position between particles, which is defined as 2*R*_p_ [52]. The wall condition of all boundaries was typically set as bounce when tracing microscopic particles in a fluid field. 

## 3. Results and Discussion 

**Figure 1:**
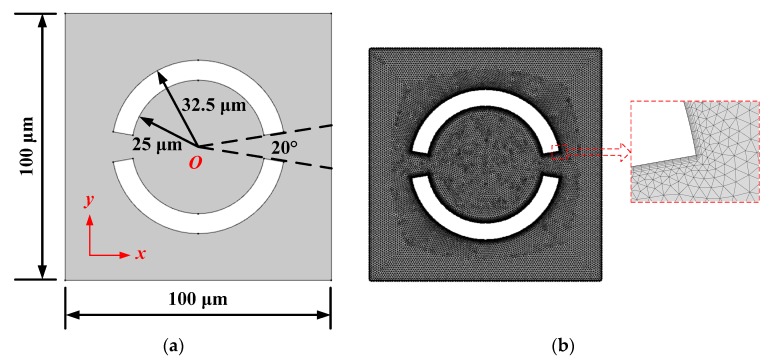

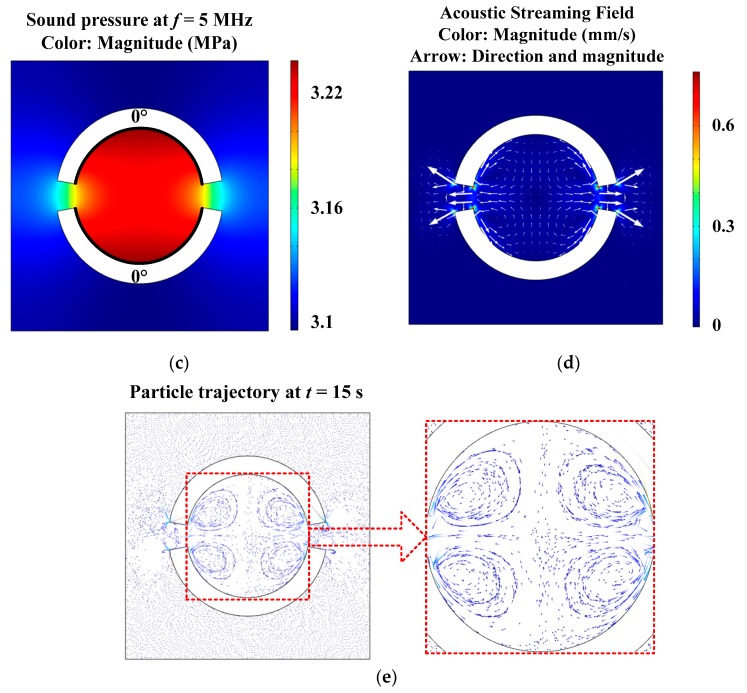
(Color online). Two-dimensional (2D) square model for the acoustofluidic field and particle trajectory excited by two vibration sources with the same frequency (5 MHz) and amplitude (1 nm). (**a**) Computational model. (**b**) Meshed model for the acoustofluidic field and particle trajectory. (**c**) Pattern of sound pressure field. (**d**) Pattern of acoustic streaming field. (**e**) Pattern of microparticle trajectory at a given time (15 s).

The acoustic streaming field actuated by two ring-shaped vibration sources in a square (*L* = 100 μm) microfludic chamber was computed to validate the simulation method, as shown in Figure 1a. The inner and outer radii of the ring-shaped vibration sources were set to be 25 and 32.5 μm, respectively. The included angle of the fan-shaped zones, represented by the dashed lines in Figure 1a is 20°, was used to represent the gap of the neighboring vibration sources. Figure 1b shows a meshed model for the acoustofluidic field with a locally enlarged view of the boundary layer. Most of the regions in Figure 1b are divided into free triangular mesh with a maximum size of 0.5 μm, which is 1/600 of the wavelength of the sound field at 5 MHz ($\lambda =\frac{c_{0}}{f}=\frac{1500\;m}{5\;\mathrm{MHz}}=300\;\mu m$). The number of the boundary layer was 5, and the thickness of first layer was manually set to be 0.05 μm, which is 1/5 of the calculated viscous boundary layer thickness in water when the input frequency is 5 MHz ($\delta =\sqrt{\frac{\mu }{\pi \rho_{0}f}}=\sqrt{\frac{0.001\;\mathrm{Pa}\cdot s}{\pi \times 1000{\;\mathrm{kg}/m}^{3}\times 5\;\mathrm{MHz}}}\approx 0.25\;\mu m$) [50]. The number of grid cells in the simulation was about 35,000. The total simulation time of the sound field and the fluid flow field was usually less than 2 min by using a DELL T5820 Tower workstation with a 128 GB memory (Dell Inc., Shanghai Subsidiary, Shanghai, China), while the trajectory simulation time of 10,000 microparticles (polystyrene beads) was several hours. It was proved that the results were converged and mesh-independent. A COMSOL simulation file of Figure 1 (Two vibration sources with the same initial phase, version 5.4) is available online as Supplementary Materials by referring to the existing simulation case on the Internet [58]. Unless otherwise specified, the properties of the vibration sources and the acoustofluidic medium (water) in the simulation are listed in Table 1.

### 3.1. Model Validation

The sound field and the acoustic streaming field, generated by two ring-shaped vibration sources, were simulated to validate the proposed model. Figure 1c shows the computed sound pressure in the square chamber generated by two radiation surfaces (represented by a thick, black, solid curve) with the same input frequency (5 MHz), radial vibration amplitude (1 nm), and initial phase (0°). The color bar in Figure 1c denotes the magnitude of the sound pressure. Figure 1d shows the simulated acoustic streaming field induced by the ultrasonic field in Figure 1c. In Figure 1d, the color denotes the magnitude of the acoustic streaming velocity, $\left\|u_{2}\right\|$, and the white arrows denote the direction and magnitude of the acoustic streaming velocity. In the microfluidic cavity, four petaloid acoustic streaming vortices can be obviously observed and adjacent vortices flow in opposite directions. In order to ensure the calculation accuracy of the particle trajectory in Figure 1e, the acoustic streaming-induced drag force and the acoustic radiation force were both considered for the 1-um-diameter particle movement simulation at 5 MHz. However, compared with the streaming-induced drag force, the influence range of acoustic radiation force was mainly around the edge of vibration sources and could sometimes be neglected [56]. More detailed description and comparisons between acoustic streaming-induced drag force and acoustic radiation force (shown in Figures S1 and S2) can be found in the Supplementary Material. The acoustic streaming pattern in Figure 1d and the simulated particle trajectory with comet tails at *t* = 15 s in Figure 1e are qualitatively consistent with the streamline of 1-μm-diameter tracer fluorescent beads in an acoustofluidic cavity, as proposed by Wiklund et al. 2012 [32]. According to the described experiment in the above-mentioned literature, this kind of microfluidic device can be used for microparticle aggregation in the cavity center. According to the simulation result of particle trajectory shown in Figure 1e, except microparticles moving with the four-petal-shaped vortex field, remaining particles can form a cross-shaped aggregation pattern in the center of the acoustofluidic cavity, which is consistent with the described experimental phenomenon. Due to the existence of sharp edges in our simulation model, localized strong streaming vortices (long white arrows in Figure 1d) originating from the structural edge oscillation are observed around the fan-shaped gaps. Thus, a part of the particles near the gaps may flow out of the cavity along with localized strong vortices. The calculated results of the acoustofluidic field and particle trajectory in Figure 1 indicate that the acoustic streaming theory and simulation results are feasible and credible.

In the following sub-sections, the proposed method is used to simulate the acoustic streaming field excited by multiple vibration sources, each of which is operated at individual initial phases. The simulation results reveal a diversity of acoustic streaming patterns only influenced by the number and oscillation condition of the vibration sources.

### 3.2. Acoustic Streaming Excited by Single-Layer Vibration Sources

#### 3.2.1. Vibration Sources with the Same Initial Phase

**Figure 2:**
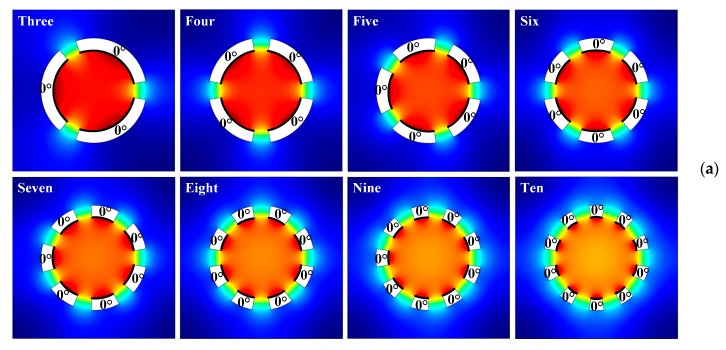

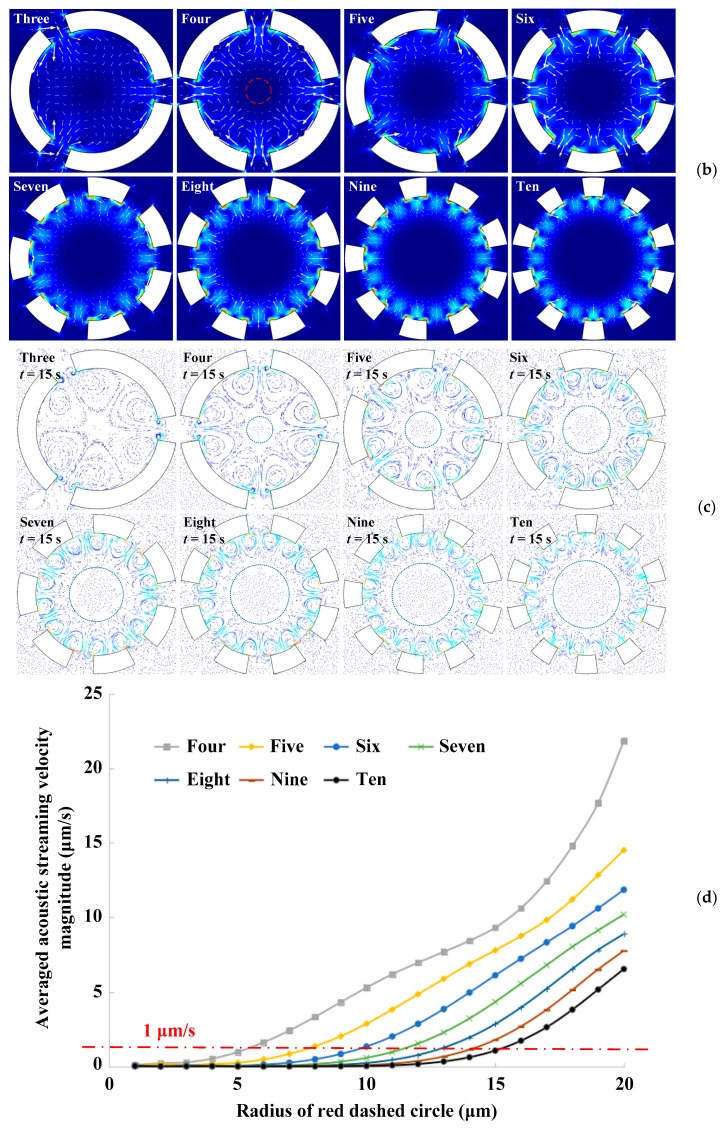

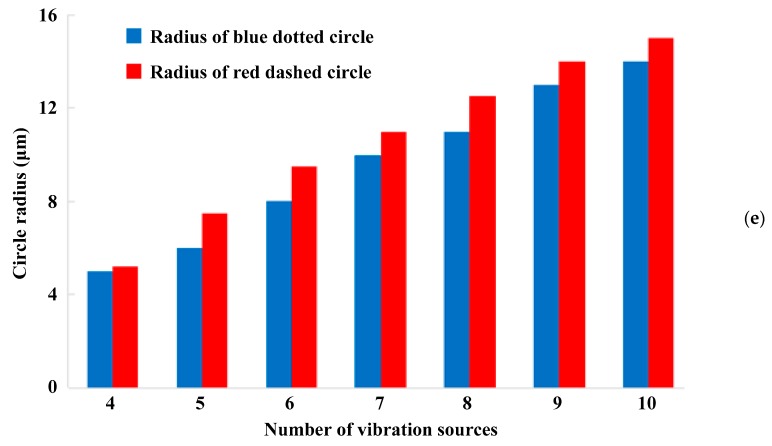
(Color online). Acoustofluidic field and particle trajectory excited by single-layer vibration sources with the same initial phase. (**a**) Patterns of sound pressure field. (**b**) Patterns of acoustic streaming field. (**c**) Patterns of microparticle trajectory. (**d**) Averaged acoustic streaming velocity magnitude vs. radius of red dashed circle. (**e**) Different circle radii vs. number of vibration sources.

On the basis of the above-mentioned two vibration sources, the number of segmented ring-shaped vibration sources increases from three to ten, while the angle of fan-shaped gap keeps constant (20°). The simulation results of the sound field, acoustic streaming, and particle trajectory at a given time generated by different numbers of vibration sources are shown in Figure 2a–c, respectively. The initial phases of all vibration sources are the same and are expressed in terms of 0° in Figure 2a. The radial amplitudes of all vibration sources are kept constant (1 nm). According to the simulation result of the acoustic streaming field shown in Figure 2b, the number of the acoustic streaming vortices is twice that of the vibration sources. Each pair of vortices with opposite directions flows out from the center of the radiation surface and flows in from the edges of the vibration source. However, due to the existence of the fan-shaped gaps, the acoustic streaming field continues to flow out from the gaps, which also can be observed from the particle trajectory simulation results in Figure 2c. For the case of three vibration sources, even after a considerable time period (*t* = 15 s), it is still difficult to stabilize microparticle concentration in the microfluidic cavity center, and most of the particles left in the cavity keep rotating with the acoustic streaming vortex field. Starting from the case of four vibration sources, the acoustic streaming field can be used to aggregate microparticles to the cavity center, and the stable patterns of microparticle aggregation are directly related to the number of vibration sources. It is observed that microparticles can form curved-edge polygons with different side numbers in the cavity center, and the side number is directly consistent with the vibration source number. It is also found that with the increase of the vibration source number, the particle aggregation area becomes larger. Considering that the radial vibration amplitudes and input frequencies of all vibration sources were kept constant in our simulation, the total radiant sound power *P*_total_ of vibration sources is proportional to the total curve length *L*_total_ of radiation surfaces, which can be expressed as [18]:

(20) $$P_{\mathrm{total}}\propto L_{\mathrm{total}}=\left(2\pi -N\theta_{f}\right)R_{i}$$

where *θ_f_* is the angle of fan-shaped gap and *N* and *R_i_* are the number and the inner radius of the vibration sources, respectively (shown in Table 1). The acoustic streaming velocity, together with the particle aggregation size, is dependent of the total radiant sound power *P*_total_. An increase of the vibration source number or the fan-shaped gap angle will result in a decrease of acoustic streaming velocity and an increase of particle aggregation area size.

In order to quantitatively describe the above-mentioned phenomenon, the relationship between the averaged acoustic streaming velocity magnitude $\frac{\oint_{R}\left\|u_{2}\right\|ds}{2\pi R}$ along the red dashed circle (shown in Figure 2b) and the circle radius *R* is analyzed with different vibration source numbers, as shown in Figure 2d. The overall tendency of all curves in Figure 2d is that with the increase of the distance to the cavity center (the circle radius *R*), the averaged acoustic streaming velocity magnitude increases in an approximate quadratic form. However, compared with the case of a small number of vibration sources, the curve slope of the averaged acoustic streaming velocity magnitude decreases significantly with the number increase of vibration sources. The distance from the apex angle to the center of the polygonal pattern (the radius of the circumscribed circle represented by the blue dotted line in Figure 2c) with different side numbers is also measured and represented by the blue histogram in Figure 2e. In order to compare this with the averaged acoustic streaming velocity magnitude, a red chain line is drawn in Figure 2d to represent the case where the averaged acoustic streaming velocity magnitude is 1 μm/s, and the corresponding radius of the red dashed circle is represented by the red histogram in Figure 2e. It can be clearly seen from Figure 2e that the size of the aggregation pattern formed by microparticles can be roughly estimated by measuring the range of the low-speed acoustic streaming field region (potential well of acoustic streaming velocity magnitude) inside the acoustofluidic cavity.

**Figure 3:**
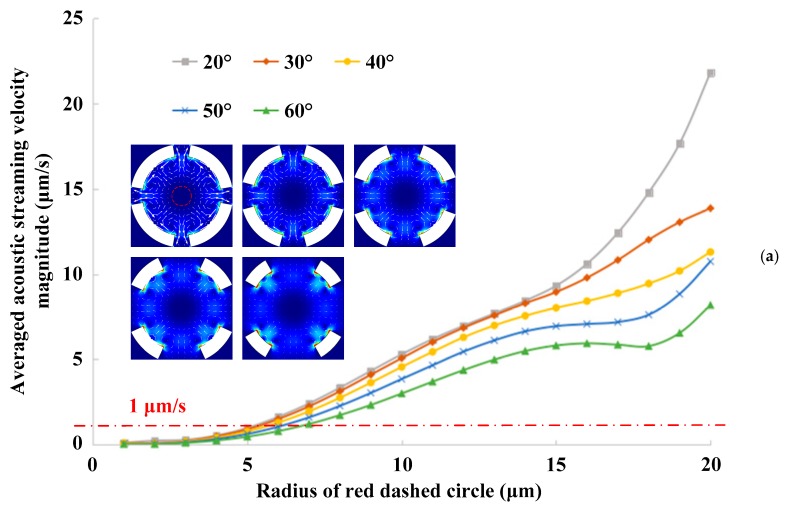

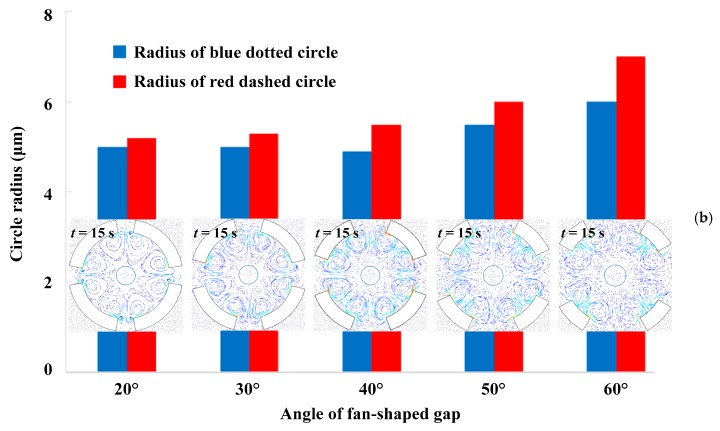
(Color online). Acoustic streaming field and particle trajectory excited by four vibration sources with the same initial phase and different fan-shaped gap angles. (**a**) Averaged acoustic streaming velocity magnitude vs. radius of red dashed circle. (**b**) Different circle radii vs. angle of fan-shaped gap.

Based on the model of four vibration sources, the influence of different fan-shaped gap angles (20°, 30°, 40°, 50°, 60°) on acoustofluidic fields and particle motion is also studied. The relationship between the averaged acoustic streaming velocity magnitude $\frac{\oint_{R}\left\|u_{2}\right\|ds}{2\pi R}$ along the red dashed circle (shown in Figure 2b) and the circle radius *R* is calculated with different fan-shaped gap angles, and the result is shown in Figure 3a. Compared with small fan-shaped gap angles, the larger the angle is, the smaller the averaged acoustic streaming velocity magnitude and the overall curve slope are, which can be explained by Equation (20). The curved-edge polygonal aggregation region of microparticles under different gap angles is also simulated by the particle tracing module, and the aggregation patterns at *t* = 15 s are shown in Figure 3b. Similar to Figure 2e, the distance from the apex angle to the center of the polygon or the circumscribed circle radius of the aggregation pattern represented by the blue dotted line is measured and represented by the blue histogram in Figure 3b. A red chain line is drawn in Figure 3a to represent the case where the averaged acoustic streaming velocity magnitude is 1 μm/s, and the corresponding radius of the red dashed circle (shown in Figure 2b) is represented by the red histogram in Figure 3b. According to the simulation results, the fan-shaped gap angle has a slight influence on the aggregation pattern size of microparticles.

In summary, multiple vibration sources working at the same initial phase can form a low-speed acoustic streaming field region (potential well of acoustic streaming velocity magnitude), which can be used to aggregate microparticles, and the stable aggregation pattern and size are dependent on the total radiant sound power relating to the number of vibration sources and the fan-shaped gap angle. With the increase of the vibration source number or the fan-shaped gap angle, the star-shaped polygonal concentration region of microparticles also becomes larger, which depends on the region of the low-speed acoustic streaming field. However, the angle of the fan-shaped gap has a minor influence on the aggregation pattern size of microparticles.

#### 3.2.2. Vibration Sources with Different Initial Phases

**Figure 4:**
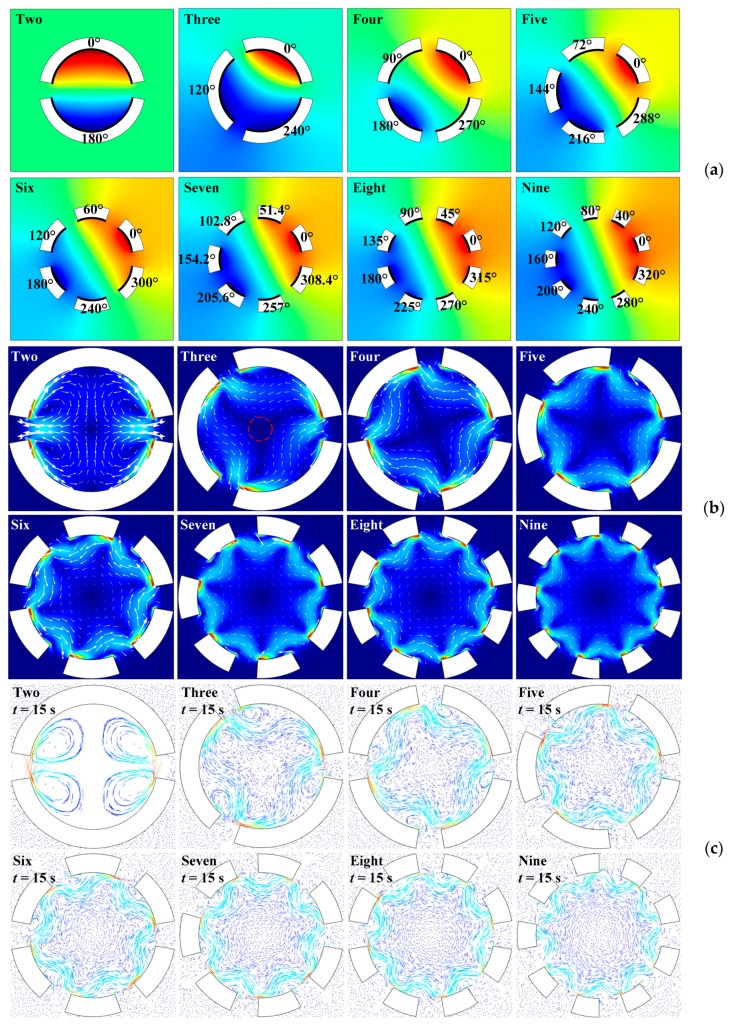

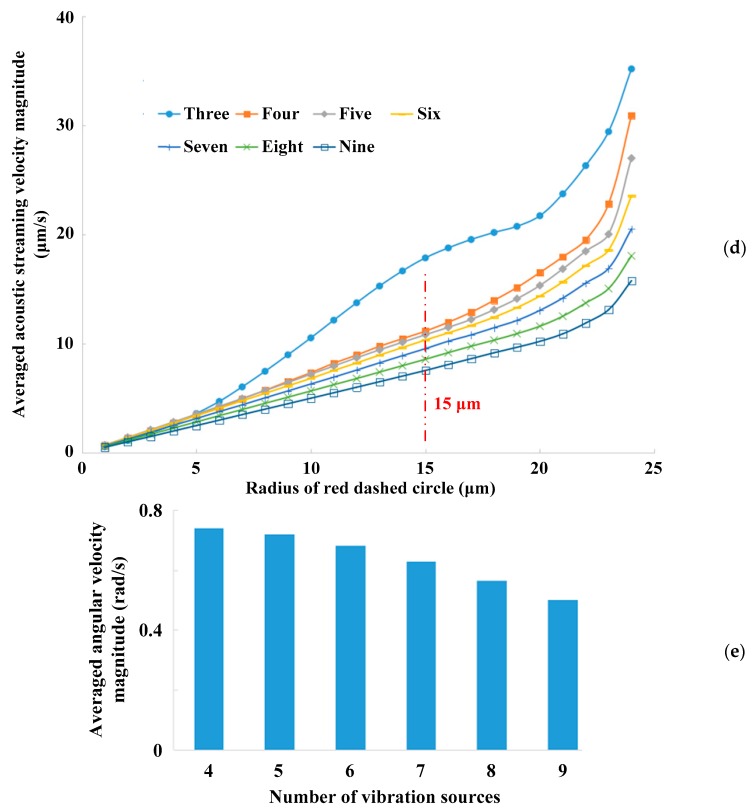
(Color online). Acoustofluidic field and particle trajectory excited by single-layer vibration sources with different initial phases. (**a**) Patterns of sound pressure field. (**b**) Patterns of acoustic streaming field. (**c**) Patterns of microparticle trajectory. (**d**) Averaged acoustic streaming velocity magnitude vs. radius of red dashed circle. (**e**) Averaged angular velocity magnitude vs. number of vibration sources.

The acoustofluidic field and particle trajectory generated by multiple vibration sources with different initial phases are simulated, and the simulation results are shown in Figure 4, while the radial oscillation amplitudes of all vibration sources are kept constant (1 nm). All patterns of the sound field distribution in Figure 4a are approximately anti-symmetric with respect to initial phases of 0° and 180°. The simulated acoustic streaming pattern of two vibration sources with different initial phases is similar to that in Figure 1d, and the flow pattern consists four petal-shaped vortices, as shown in Figure 4b. The maximum acoustic streaming velocity magnitude generated by two vibration sources with the same initial phase is about 7 × 10^−4^ m/s, as shown in Figure 1d, while the maximum acoustic streaming velocity magnitude generated by two vibration sources with different initial phases (0° and 180°) is about 2.5 × 10^−3^ m/s. Therefore, for the case of two vibration sources, although the acoustic streaming field distribution cannot be obviously changed by tuning initial phases, the acoustic streaming velocity magnitude can be modulated. According to the particle trajectory simulation result of two vibration sources in Figure 4c, due to the existence of strong outward vortices from the fan-shaped gaps, only a small number of microparticles can be left in the microfluidic cavity and rotated rapidly with the acoustic streaming vortices. Thus, the acoustofluidic cavity composed of two vibration sources with opposite initial phases can be used for particle removal and rapid microfluidic mixing.

Starting from the case of three vibration sources in Figure 4b, the acoustic streaming field generated by multiple vibration sources with different initial phases contains one main circumferential vortex inside the acoustofluidic cavity. The vortex rotation direction is consistent with the increasing direction of the initial phases, and this is because the vibration source of earlier excitation will push the fluid medium from the radiation surface to that of later excitation. Since the initial phase distribution increases along the counterclockwise direction in our simulation, the vortex rotation direction is also counterclockwise. The relationship between the averaged acoustic streaming velocity magnitude $\frac{\oint_{R}\left\|u_{2}\right\|ds}{2\pi R}$ along the red dashed circle (shown in Figure 4b) and the circle radius *R* is also calculated with different vibration sources, which is shown in Figure 4d. Starting from the case of four vibration sources, the curves of the averaged acoustic streaming velocity magnitude are approximately linear with the radius to the cavity center before the radius is 15 μm (represented by the red chain line in Figure 4d), and the curve slope representing the averaged angular velocity magnitude $\frac{\oint_{R}\left\|u_{2}\right\|ds}{2\pi R^{2}}$ of the low-speed acoustic streaming field decreases with the increase of the vibration source number, which is represented by the histogram in Figure 4e.

Starting from the case of three vibration sources, due to the existence of the low-speed acoustic streaming field region with circumferential rotating component, microparticles aggregated in the center of the acoustofluidic cavity remain stationary, while other particles near the radiation surfaces of the vibration sources rotate in the circumferential direction, and the rotation trajectory is consistent with the high-speed circumferential flow fluid in the cavity, as shown in Figure 4c. Compared with the simulation results in Figure 2c, all of the aggregation patterns formed by microparticles in Figure 4c are approximately circular, and all radii of the patterns are about 4 μm.

In summary, multiple vibration sources working at different initial phases can form rotational acoustic streaming fields. Starting from three vibration sources, a circumferential rotating vortex field can be generated in the microfluidic cavity, and the distribution of the acoustic streaming vortex satisfies that the closer to the cavity center, the smaller the acoustic streaming velocity magnitude is. Thus, acoustofluidic cavity consisting multiple vibration sources with different initial phases can also be used to aggregate microparticles in the cavity center together with the functions, including microfluidic stirring and particle mixing.

### 3.3. Acoustic Streaming Excited by Double-Layer Vibration Sources

The diversity of the acoustic streaming pattern is directly related to the number, distribution, initial phases, and other structure and vibration parameters of multiple vibration sources. On the basis of single-layer vibration sources, the structural design of double-layer vibration sources by referring to the style of a Russian doll or Matryoshka is also investigated [45]. Preliminary investigation of simple Russian doll- or Matryoshka-type configurations (double-layer vibration sources) can provide a novel method of multifarious structure design in following research on the combination of phononic crystals and acoustic streaming fields, which can provide more abundant structural design ideas for acoustofluidic devices.

**Figure 5:**
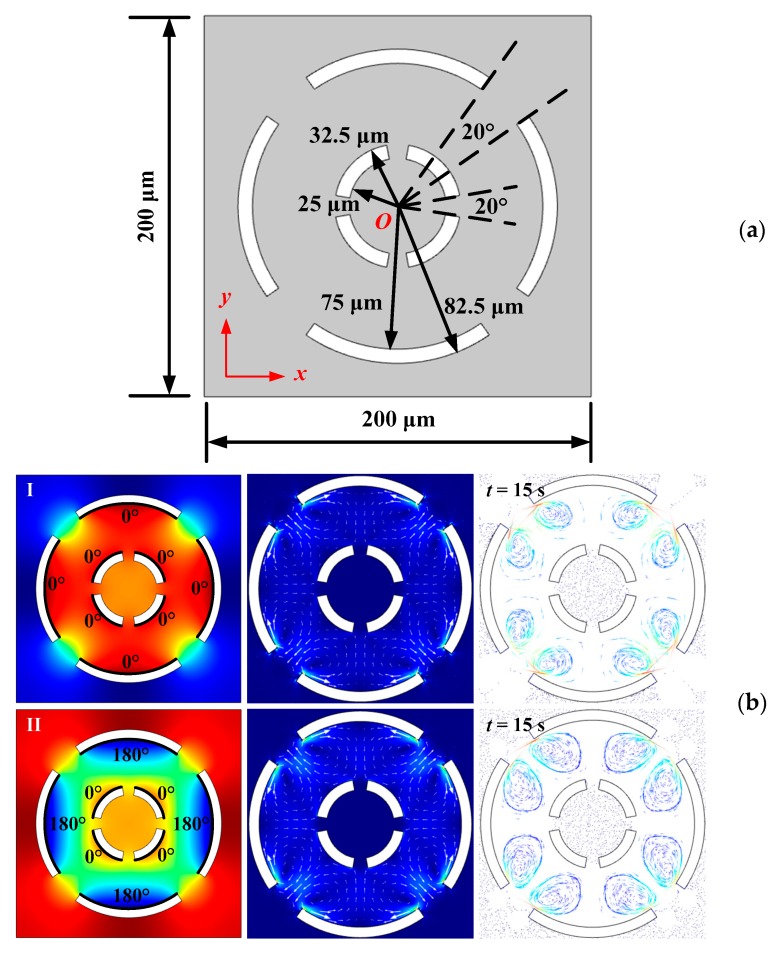

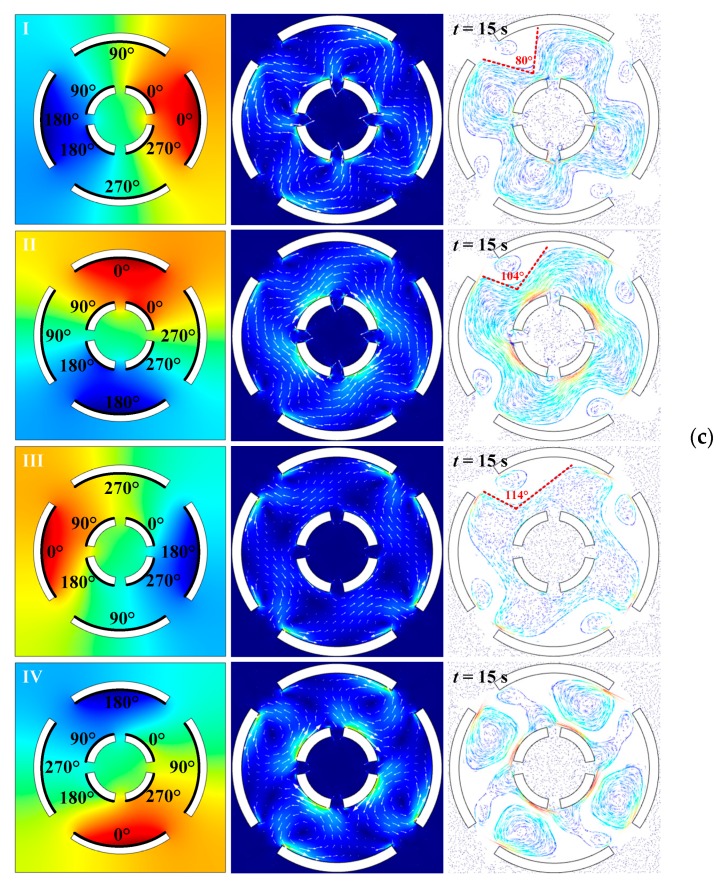
(Color online). Acoustofluidic field and particle trajectory excited by double-layer vibration sources with different initial phases. (**a**) Computational model. (**b**) Simulation results with the same initial phase of the identical layer. (**c**) Simulation results with different initial phases of the identical layer.

The distribution and corresponding size of the double-layer vibration sources are shown in Figure 5a. The numbers of the vibration sources located in the inner and outer layers are both set to be four. The inner-layer and outer-layer vibration sources are staggered by 45° in space. The size of inner-layer vibration sources is the same as before, while the inner and outer radii of the outer-layer ring-shaped vibration sources are set as 75 and 82.5 μm, respectively. In order to accommodate the double-layer vibration sources, the side length of the square microfluidic chamber is increased to 200 μm. The angle of the fan-shaped gaps represented by the dashed line in Figure 5a is still set to be 20°, which is used to represent the interval angle of neighboring vibration sources.

According to the above-mentioned calculation results, the sound field and acoustic streaming field generated by the radiation surfaces are mainly restricted in the acoustofluidic cavity enclosed by the ring-shaped vibration sources. As shown in Figure 5b,c, the outer side of the inner-layer vibration sources and the inner side of the outer-layer vibration sources are set as the radiation surfaces of the double-layer vibration source configuration. Due to the existence of eight radiation surfaces with individual initial phases, the optional initial phase distribution of the double-layer vibration sources is abundant. Two types of initial phase distribution are considered: One is that the initial phase distribution of the identical layer of vibration sources is the same (shown in Figure 5b), and the other is that the initial phase distribution of the identical layer of vibration sources is set from 0° to 270° (shown in Figure 5c). The radial oscillation amplitudes of all vibration sources are kept constant (1 nm). As the radiation surface length of the outer-layer vibration sources is approximately three times that of the inner-layer vibration sources, the acoustic streaming field in the microfluidic cavity is mainly dependent on the initial phase distribution of the outer-layer vibration sources (see Equation (20)). According to our previous calculation results shown in Figure 2b and Figure 4b, the low-speed acoustic streaming field region (potential well of acoustic streaming velocity magnitude) generated by the outer-layer vibration sources is located in the acoustofluidic cavity center. Therefore, the existence of the inner-layer vibration sources also has local influence on the acoustic streaming vortices generated by the outer-layer vibration sources. Detailed analyses of the sound field, acoustic streaming, and particle trajectory generated by the double-layer vibration sources in Figure 5b,c are described as follows.

As shown in Figure 5b, Row I represents that all the initial phases of the inner- and outer-layer vibration sources are the same, while Row II represents that the initial phase difference between the inner- and outer-layer vibration sources is 180°. However, the acoustic streaming field distribution generated by these two situations is approximately the same, consisting of eight main vortices in the microfluidic cavity composed of double-layer vibration sources. Since the inner- and outer-layer vibration sources are staggered by 45° in space, each acoustic streaming vortex flows out from the radiation surface of the vibration sources and flows in from the fan-shaped gap between the neighboring vibration sources. The maximum acoustic streaming velocity magnitude in Row I is about 3.5 × 10^−4^ m/s, while the maximum acoustic streaming velocity magnitude in Row II is about 1.6 × 10^−4^ m/s. Although the calculated acoustic streaming distribution cannot be used to distinguish these two situations, qualitative analysis of particle trajectory simulation can be conducted. Compared with the case that all vibration sources have the same initial phase, the rotation region of microparticles generated by different initial phases between the inner- and outer-layer vibration sources is larger and clearer at the boundaries.

Figure 5c shows a more complex initial phase distribution of double-layer vibration sources, while the sound fields, acoustic streaming patterns, and particle trajectories are more diverse. For the identical layer of vibration sources, the distribution of the initial phases increases from 0° to 270° along the counterclockwise direction. However, the vibration sources with the same initial phase of the two layers can be staggered with different angles in space, so there are altogether four distribution situations, as shown in Figure 5c. The maximum acoustic streaming velocity magnitudes from Row I to Row IV in Figure 5c are 2.6 × 10^−4^, 2.7 × 10^−4^, 1.6 × 10^−4^, and 1.7 × 10^−4^ m/s, respectively. Taking the inner-layer vibration source with the initial phase of 0° as a reference, the outer-layer vibration source with the same initial phase is staggered from −45° to 215° in space from Row I to Row IV, which can be defined as *θ*. It is found that with the increase of *θ*, distortion of the acoustic streaming pattern and fragmentation of local vortex field occurs, and a similar conclusion can also be drawn from the particle trajectory patterns from Row I to Row IV. In order to quantitatively characterize the distortion degree of the acoustic streaming field, the included angles between two neighboring curves of the particle trajectory patterns from Row I to Row III are represented by two red dashed lines, and the measured angle value is approximately 80°, 104°, and 114°, respectively. Further increase of *θ* eventually leads to fragmentation and separation of the acoustic streaming vortex field, as shown in Row IV.

In summary, the Matryoshka-type acoustofluidic chamber, which consists of double-layer vibration sources working at different initial phases, can form diverse acoustic streaming vortices for particle rotation and microfluidic mixing. Distortion and fragmentation of acoustic streaming patterns can be used to study the formation mechanism of microfluidic vortices. Although the increase in the number of small eddies is beneficial to mixing, too many vortices produced by multiple acoustic transducers may also lead to a decrease in the vortex intensity [40]. The arrangement of multiple vibration sources with individual oscillation phases may need to be modulated for a better mixing effect. 

## 4. Conclusions

In this paper, we have numerically computed and analyzed the two-dimensional acoustic streaming fields in an ultrasonic cavity, consisting of multiple segmented ring-shaped vibration sources. The computational result agrees well with the existing experimental observation, and diverse acoustic streaming patterns are easily available by modifying the initial phases of different vibration sources which only vibrate along the radial direction. The particle trajectory simulation result demonstrates that the proposed acoustofluidic cavity can be used for simultaneous concentration and rotation of microparticles. It is found that the shape and size of particle concentration patterns are directly consistent with the range of low-speed acoustic streaming field regions (potential well of acoustic streaming velocity magnitude) inside the acoustofluidic cavity. Preliminary investigation of simple Russian doll- or Matryoshka-type configurations provide a novel method of diverse structure design in future researches on the combination of sonic crystal and acoustic streaming. The implementation of multiple vibration sources offers flexibility for the control of acoustic streaming fields in microfluidic devices for various applications, which is expected to be a promising tool for the investigation of rapid microfluidic mixing on a chips and contactless rotational manipulation of trapped biosamples, such as cells or nematodes for morphology observation and phenotype characterization.

## Figures and Tables

**Table 1:** Model parameters in the simulation.

Quantity	Abbreviation	Value	Unit
Side length of square chamber	*L*	100	μm
Inner radius of vibration source	*R_i_*	25	μm
Outer radius of vibration source	*R_o_*	32.5	μm
Angle of fan-shaped gap	*θ_f_*	20	°
Radial vibration amplitude	*A_r_*	1	nm
Input vibration frequency	*f*	5	MHz
Density of water	*ρ*_0_	1000	kg/m^3^
Speed of sound in water	*c*_0_	1500	m/s
Shear viscosity of water	*μ*	0.001	Pa·s
Volume-to-shear viscosity ratio in water	*μ_B_*/*μ*	2.79	1
Heat capacity at constant pressure of water	*C_P_*	4200	J/(kg·K)
Heat conductivity coefficient of water	*k*	0.6	W/(m·K)
Density of microparticle	*ρ*_p_	1050	kg/m^3^
Speed of sound in microparticle	*c*_p_	2400	m/s
Diameter of microparticle	*D*_p_	1	μm
Spring constant of polystyrene bead	*k_s_*	2.5 × 10^−4^	N/m

## Supplementary Materials

The following are available online at https://www.mdpi.com/2072-666X/10/12/803/s1, Figure S1: (Color online). Acoustofluidic field excited by four vibration sources with the same initial phase. (a) Acoustic radiation force magnitude. (b) Acoustic streaming induced starting drag force magnitude, Figure S2: (Color online). Acoustofluidic field excited by four vibration sources with different initial phases. (a) Acoustic radiation force magnitude. (b) Acoustic streaming induced starting drag force magnitude, together with a COMSOL simulation file of Figure 1.

## References

[1] Bruus H., Dual J., Hawkes J., Hill M., Laurell T., Nilsson J., Radel S., Sadhal S., Wiklund M. (2011). Forthcoming Lab on a Chip tutorial series on acoustofluidics: Acoustofluidics-exploiting ultrasonic standing wave forces and acoustic streaming in microfluidic systems for cell and particle manipulation. Lab Chip.

[2] Friend J., Yeo L.Y. (2011). Microscale acoustofluidics: Microfluidics driven via acoustics and ultrasonics. Rev. Mod. Phys..

[3] Whitesides G.M. (2006). The origins and the future of microfluidics. Nature.

[4] Baresch D., Thomas J.L., Marchiano R. (2016). Observation of a single-beam gradient force acoustical trap for elastic particles: Acoustical tweezers. Phys. Rev. Lett..

[5] Marston P.L. (2006). Axial radiation force of a Bessel beam on a sphere and direction reversal of the force. J. Acoust. Soc. Am..

[6] Lu X., Shen H., Zhao K., Wang Z., Peng H., Liu W. (2019). Micro/nano machines driven by ultrasound power sources. Chem. Asian J..

[7] Gong Z., Marston P.L., Li W. (2019). Reversals of acoustic radiation torque in Bessel beams using theoretical and numerical implementations in three dimensions. Phys. Rev. Appl..

[8] Wu M., Ozcelik A., Rufo J., Wang Z., Fang R., Huang T.J. (2019). Acoustofluidic separation of cells and particles. Microsyst. Nanoeng..

[9] Meng L., Cai F., Li F., Zhou W., Niu L., Zheng H. (2019). Acoustic tweezers. J. Phys. D Appl. Phys..

[10] Nilsson J., Evander M., Hammarström B., Laurell T. (2009). Review of cell and particle trapping in microfluidic systems. Anal. Chim. Acta.

[11] Andersson H., Berg A.V.D. (2003). Microfluidic devices for cellomics: A review. Sens. Actuators B Chem..

[12] Cui P., Wang S. (2019). Application of microfluidic chip technology in pharmaceutical analysis: A review. J. Pharm. Anal..

[13] Pratt E.D., Huang C., Hawkins B.G., Gleghorn J.P., Kirby B.J. (2011). Rare cell capture in microfluidic devices. Chem. Eng. Sci..

[14] Baudoin M., Thomas J.L. (2020). Acoustic tweezers for particle and fluid micromanipulation. Annu. Rev. Fluid Mech..

[15] Erickson D., Li D. (2004). Integrated microfluidic devices. Anal. Chim. Acta.

[16] Yeo L.Y., Friend J.R. (2014). Surface Acoustic Wave Microfluidics. Annu. Rev. Fluid Mech..

[17] Iliescu C., Tresset G., Xu G. (2009). Dielectrophoretic field-flow method for separating particle populations in a chip with asymmetric electrodes. Biomicrofluidics.

[18] Hu J. (2014). Ultrasonic Micro/Nano Manipulations: Principles and Examples.

[19] Zhou T., Deng Y., Zhao H., Zhang X., Shi L., Joo S.W. (2018). The mechanism of size-based particle separation by dielectrophoresis in the viscoelastic flows. J. Fluids Eng..

[20] Reverté L., Prieto-Simón B., Campàs M. (2016). New advances in electrochemical biosensors for the detection of toxins: Nanomaterials, magnetic beads and microfluidics systems. A review. Anal. Chim. Acta.

[21] Kotz K.T., Noble K.A., Faris G.W. (2004). Optical microfluidics. Appl. Phys. Lett..

[22] Luong T.D., Nguyen N.T. (2010). Surface acoustic wave driven microfluidics-a review. Micro Nanosyst..

[23] Yamada M., Seki M. (2005). Hydrodynamic filtration for on-chip particle concentration and classification utilizing microfluidics. Lab Chip.

[24] Di Carlo D. (2009). Inertial microfluidics. Lab Chip.

[25] Zhou T., Xu Y., Liu Z., Joo S.W. (2015). An enhanced one-layer passive microfluidic mixer with an optimized lateral structure with the Dean effect. J. Fluids Eng..

[26] Shi J., Ahmed D., Mao X., Lin S.C.S., Lawit A., Huang T.J. (2009). Acoustic tweezers: Patterning cells and microparticles using standing surface acoustic waves (SSAW). Lab Chip.

[27] Marston P.L., Thiessen D.B. (2004). Manipulation of fluid objects with acoustic radiation pressure. Ann. N. Y. Acad. Sci..

[28] Bernassau A.L., Glynne-Jones P., Gesellchen F., Riehle M., Hill M., Cumming D.R. (2014). Controlling acoustic streaming in an ultrasonic heptagonal tweezers with application to cell manipulation. Ultrasonics.

[29] Connacher W., Zhang N., Huang A., Mei J., Zhang S., Gopesh T., Friend J. (2018). Micro/nano acoustofluidics: Materials, phenomena, design, devices, and applications. Lab Chip.

[30] Rudenko O.V., Sarvazyan A.P., Emelianov S.Y. (1996). Acoustic radiation force and streaming induced by focused nonlinear ultrasound in a dissipative medium. J. Acoust. Soc. Am..

[31] Liu S., Yang Y., Ni Z., Guo X., Luo L., Tu J., Zhang D., Zhang A.J. (2017). Investigation into the effect of acoustic radiation force and acoustic streaming on particle patterning in acoustic standing wave fields. Sensors.

[32] Wiklund M., Green R., Ohlin M. (2012). Acoustofluidics 14: Applications of acoustic streaming in microfluidic devices. Lab Chip.

[33] Muller P.B., Barnkob R., Jensen M.J., Bruus H. (2012). A numerical study of microparticle acoustophoresis driven by acoustic radiation forces and streaming-induced drag forces. Lab Chip.

[34] Riaud A., Baudoin M., Thomas J.L., Bou Matar O. (2014). Cyclones and attractive streaming generated by acoustical vortices. Phys. Rev. E.

[35] Riaud A., Baudoin M., Bou Matar O., Thomas J.L., Brunet P. (2017). On the influence of viscosity and caustics on acoustic streaming in sessile droplets: An experimental and a numerical study with a cost-effective method. J. Fluid Mech..

[36] Bach J.S., Bruus H. (2019). Bulk-driven acoustic streaming at resonance in closed microcavities. Phys. Rev. E.

[37] Tang Q., Hu J. (2015). Diversity of acoustic streaming in a rectangular acoustofluidic field. Ultrasonics.

[38] Lu X., Soto F., Li J., Li T., Liang Y., Wang J. (2017). Topographical manipulation of microparticles and cells with acoustic microstreaming. ACS Appl. Mater. Interfaces.

[39] Dentry M.B., Yeo L.Y., Friend J.R. (2014). Frequency effects on the scale and behavior of acoustic streaming. Phys. Rev. E.

[40] Tang Q., Hu J., Qian S., Zhang X. (2017). Eckart acoustic streaming in a heptagonal chamber by multiple acoustic transducers. Microfluid. Nanofluid..

[41] Marmottant P., Versluis M., de Jong N., Hilgenfeldt S., Lohse D. (2006). High-speed imaging of an ultrasound-driven bubble in contact with a wall: “Narcissus” effect and resolved acoustic streaming. Exp. Fluids.

[42] Nama N., Huang P.H., Huang T.J., Costanzo F. (2014). Investigation of acoustic streaming patterns around oscillating sharp edges. Lab Chip.

[43] Collins D.J., Ma Z., Ai Y. (2016). Highly localized acoustic streaming and size-selective sub-micron particle concentration using high frequency microscale focused acoustic fields. Anal. Chem..

[44] Courtney C.R.P., Drinkwater B.W., Demore C.E.M., Cochran S., Grinenko A., Wilcox P.D. (2013). Dexterous manipulation of microparticles using bessel-function acoustic pressure fields. Appl. Phys. Lett..

[45] Elford D.P., Chalmers L., Kusmartsev F.V., Swallowe G.M. (2011). Matryoshka locally resonant sonic crystal. J. Acoust. Soc. Am..

[46] Lighthill S.J. (1978). Acoustic streaming. J. Sound Vib..

[47] Ahmed D., Mao X., Juluri B.K., Huang T.J. (2009). A fast microfluidic mixer based on acoustically driven sidewall-trapped microbubbles. Microfluid. Nanofluid..

[48] Lamprecht A., Schwarz T., Wang J., Dual J. (2016). Acoustic radiation and viscous torque for micromanipulation controlled rotation of particles in fluid cavities. J. Acoust. Soc. Am..

[49] Ding X., Lin S.C., Kiraly B., Yue H., Li S., Chiang I.K., Shi J., Benkovic S.J., Huang T.J. (2012). On-chip manipulation of single microparticles, cells, and organisms using surface acoustic waves. Proc. Natl. Acad. Sci. USA.

[50] Bruus H. (2012). Acoustofluidics 2: Perturbation theory and ultrasound resonance modes. Lab Chip.

[51] Sadhal S.S. (2012). Acoustofluidics 13: Analysis of acoustic streaming by perturbation methods. Lab Chip.

[52] Lei J., Glynne-Jones P., Hill M. (2017). Comparing methods for the modelling of boundary-driven streaming in acoustofluidic devices. Microfluid. Nanofluid..

[53] Lei J., Hill M., Ponce de León C., Glynne-Jones P. (2018). Effects of micron scale surface profiles on acoustic streaming. Microfluid. Nanofluid..

[54] Karlsen J.T., Bruus H. (2015). Forces acting on a small particle in an acoustical field in a thermoviscous fluid. Phys. Rev. E.

[55] Tang Q., Wang X., Hu J. (2017). Nano concentration by acoustically generated complex spiral vortex field. Appl. Phys. Lett..

[56] Tang Q., Liu P., Hu J. (2018). Analyses of acoustofluidic field in ultrasonic needle-liquid-substrate system for micro-/nanoscale material concentration. Microfluid. Nanofluid..

[57] Lei J. (2017). Formation of inverse Chladni patterns in liquids at microscale: Roles of acoustic radiation and streaming-induced drag forces. Microfluid. Nanofluid..

[58] Acoustic Streaming in a Microchannel Cross Section. https://uk.comsol.com/model/acoustic-streaming-in-a-microchannel-cross-section-17087.

