# Supplementary material for: Diversity of 2D Acoustofluidic Fields in an Ultrasonic Cavity Generated by Multiple Vibration Sources
Source: Micromachines (Basel). 2019 Nov 22;10(12):803. doi: 10.3390/mi10120803 (PMC6952793; doi:10.3390/mi10120803)
Supplement: Supplementary file 1 [file micromachines-10-00803-s001.pdf]

micromachines

MDPI

Article

Supplementary Material: Diversity of 2D Acoustofluidic Fields in an Ultrasonic Cavity Generated by Multiple Vibration Sources

Qiang Tang [1,*], Song Zhou [1], Liang Huang [2] and Zhong Chen [1]

[1] Faculty of Mechanical and Material Engineering, Huaiyin Institute of Technology, Huaian 223001, China; zs41080218@126.com (S.Z.); chenzhong@hyit.edu.cn (Z.C.)

[2] School of Instrument Science and Opto-Electronics Engineering, Hefei University of Technology, Hefei 230009, China; lianghuang@hfut.edu.cn

* Correspondence: tangqiang102@126.com

1. Comparison Between Acoustic Streaming Induced Drag Forces and Acoustic Radiation Forces

From Equations (14) and (15), the acoustic radiation force magnitude can be calculated by $\left\|\mathbf{F}_{\text{rad}}\right\| = \sqrt{(F_{\text{rad}}^x)^2 + (F_{\text{rad}}^y)^2} = \frac{4}{3}\pi R_p^3 \left\|\nabla[\frac{1-\beta}{2\rho_0 c_0^2}\overline{p_1^2} - \frac{D}{2}\rho_0 \overline{\left\|\mathbf{u}_1\right\|^2}]\right\|$, and the acoustic streaming induced starting drag force magnitude can be calculated by $\left\|\mathbf{F}_{\text{drag}}\right\| = \sqrt{(F_{\text{drag}}^x)^2 + (F_{\text{drag}}^x)^2} = 6\pi\mu R_{\text{p}}\left\|\mathbf{u}_2\right\|$ [S1]. By using the post-processing functions of COMSOL Multiphysics, we can plot the patterns of the acoustic radiation force magnitude and the acoustic streaming induced starting drag force magnitude in Figure 2 (four vibration sources with the same initial phase), as shown in Figure S1.

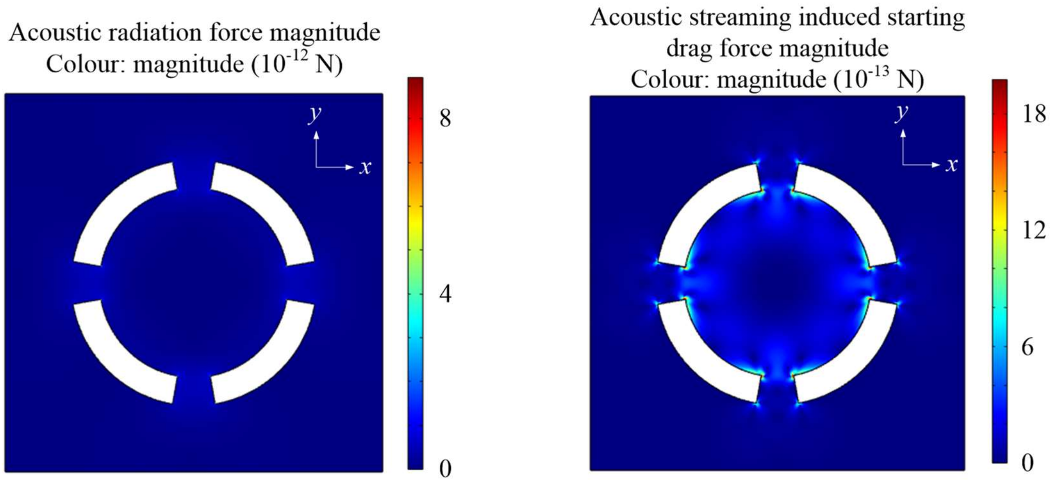

(**a**) (**b**)

Figure S1. (Color online). Acoustofluidic field excited by four vibration sources with the same initial phase. (**a**) Acoustic radiation force magnitude. (**b**) Acoustic streaming induced starting drag force magnitude.

For the case of four vibration sources with the same initial phase, the maximum acoustic streaming induced drag force magnitude is about 1.8×10^{-12} N, while the maximum radiation force magnitude is about 8×10^{-12} N, and that is the reason why micro particles can rotate with acoustic streaming vortices and aggregate in the cavity center simultaneously.

Also, we can plot the patterns of the acoustic radiation force magnitude and the acoustic streaming induced starting drag force magnitude in Figure 4 (four vibration sources with different initial phases), as shown in Figure S2.

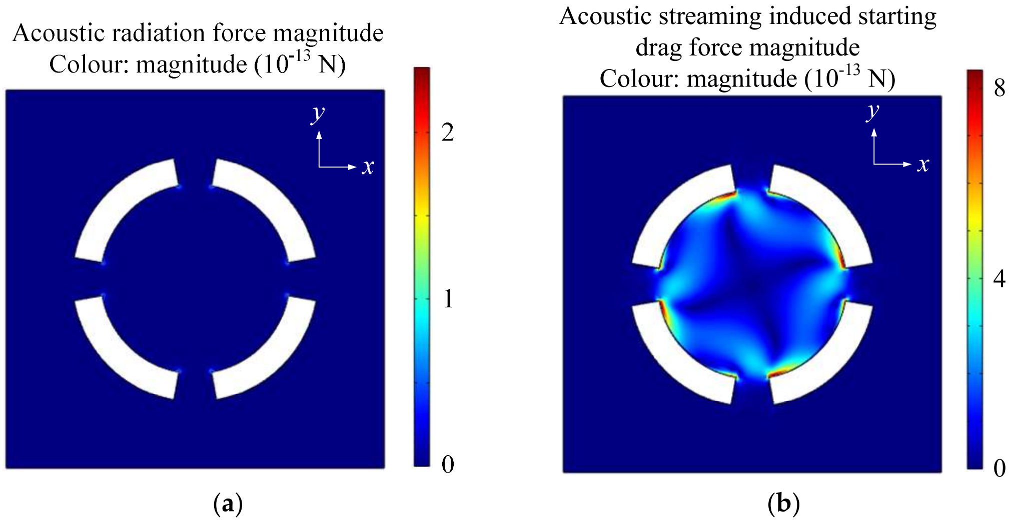

Figure S2. (Color online). Acoustofluidic field excited by four vibration sources with different initial phases. (**a**) Acoustic radiation force magnitude. (**b**) Acoustic streaming induced starting drag force magnitude.

In summary, the streaming-induced drag force and the radiation force are both needed in the calculation of 1-um-diameter particle trajectory simulation at 5 MHz. However, compared with the streaming-induced drag force, the influence range of acoustic radiation force is mainly around the edge of vibration sources and sometimes can be neglected. In order to ensure the accuracy of calculation and the completeness of theory, both acoustic streaming induced drag forces and acoustic radiation forces were considered in our particle trajectory simulation.

2. COMSOL File

A COMSOL simulation file of Figure 1 (Two vibration sources with the same initial phase, version 5.4) is available online: https://pan.baidu.com/s/1Rnc6kec-cDI2iNvJhjtUnQ (extraction code is x1zy).

Reference

S1. Tang, Q.; Liu, P.; Hu, J. Analyses of acoustofluidic field in ultrasonic needle-liquid-substrate system for micro-/nanoscale material concentration. *Microfluid. Nanofluid.* **2018**, *22*, 46.
